# Evaluating a novel approach to stimulate open science collaborations: a case series of “study-a-thon” events within the OHDSI and European IMI communities

**DOI:** 10.1093/jamiaopen/ooac100

**Published:** 2022-11-17

**Authors:** N Hughes, P R Rijnbeek, K van Bochove, T Duarte-Salles, C Steinbeisser, D Vizcaya, D Prieto-Alhambra, P Ryan

**Affiliations:** Epidemiology, Janssen R&D, Beerse, Belgium; Department of Medical Informatics, Erasmus University Medical Center, Rotterdam, The Netherlands; The Hyve, Utrecht, The Netherlands; Fundació Institut Universitari per a la recerca a l’Atenció Primària de Salut Jordi Gol i Gurina (IDIAPJGol), Barcelona, Spain; Steinbeisser Project Management, Munich, Germany; Bayer Pharmaceuticals, Sant Joan Despi, Spain; NDORMS, University of Oxford, Oxford, UK; Epidemiology, Janssen R&D, Titusville, New Jersey, USA

**Keywords:** epidemiology, clinical research, observational research, research process, data sciences

## Abstract

**Objective:**

We introduce and review the concept of a study-a-thon as a catalyst for open science in medicine, utilizing harmonized real world, observation health data, tools, skills, and methods to conduct network studies, generating insights for those wishing to use study-a-thons for future research.

**Materials and Methods:**

A series of historical study-a-thons since 2017 to present were reviewed for thematic insights as to the opportunity to accelerate the research method to conduct studies across therapeutic areas. Review of publications and experience of the authors generated insights to illustrate the conduct of study-a-thons, key learning, and direction for those wishing to conduct future such study-a-thons.

**Results:**

A review of six study-a-thons have provided insights into their scientific impact, and 13 areas of insights for those wishing to conduct future study-a-thons. Defining aspects of the study-a-thon method for rapid, collaborative research through network studies reinforce the need to clear scientific rationale, tools, skills, and methods being collaboratively to conduct a focused study. Well-characterized preparatory, execution and postevent phases, coalescing skills, experience, data, clinical input (ensuring representative clinical context to the research query), and well-defined, logical steps in conducting research via the study-a-thon method are critical.

**Conclusions:**

A study-a-thon is a focused multiday research event generating reliable evidence on a specific medical topic across different countries and health systems. In a study-a-thon, a multidisciplinary team collaborate to create an accelerated contribution to scientific evidence and clinical practice. It critically accelerates the research process, without inhibiting the quality of the research output and evidence generation, through a reproducible process.

## INTRODUCTION

Research is typically conducted in a number of key steps, over a period of time for it to be carefully conducted, within appropriate ethics and governance, and to be reproducible. This process has been refined over centuries, but within the latter 20th century, and certainly, the 21st century, the pace of research, innovation, and change has created pressure on researchers to be able to generate evidence rapidly, further exacerbated by the pace of development and expectations in health care, inclusive of the sheer volume of information on the internet.^1^ In particular, in the health domain and therapeutics, from translational discovery research to postauthorization observational research, the expectation for answers to be available today, and not in several tomorrows, has been a significant challenge.^2^

Meanwhile, the quality of research has been under considerable scrutiny, with concerns around reproducibility, rigor, and reliability of peer review, and financial motivation. In 2020, the first year of the SARS-COV-2 and Coronavirus disease 2019 (COVID-19) pandemic, it has been most evident in health care that the right data are often not in the right place to answer the right question at the right time. This exacerbates the challenge from a policy to clinical decision-making level.^3^

In response to the recognized challenges with generating reliable evidence from real-world data (RWD), collected during routine care, the Observational Medical Outcomes Partnership (OMOP) was established as a public–private partnership to study the appropriate use of observational healthcare databases for studying the effect of medicinal products.^4^ A pivotal development from this project was the OMOP common data model (CDM), which facilitated harmonization of diverse data in a standardized structure. The Observational Health Data Sciences and Informatics (OHDSI, pronounced as “Odyssey”) collaboration continued to expand the CDM by further developing standardized open-source analytical tools and best practices and creating an open science community to generate reliable federated evidence that promotes better health decisions and care.^4^

Today, the OHDSI community has in excess of 2300 registered collaborators, with more than 300 databases collectively representing over two billion patient records harmonized to the OMOP CDM.^4^ Moreover, the OHDSI network has been conducting diverse international observational health research with RWD via the CDM and analytical tools, both adding to quality science, whilst refining methodology, tools, and skills. The community has produced real-world evidence across a range of clinical areas, including cardiovascular, metabolic, immunology, oncology, and infectious diseases, inclusive of COVID-19-related research during the pandemic.^5–8^

A recent innovation, coalescing all the experience to date, tools, methods, skills, data, and clinical insights has been the “study-a-thon,” an organized event characterized by an acceleration of the research process, whilst maintaining the quality of, and confidence in, research outputs. This perspective will describe the study-a-thon in more depth, with proposed recommendations for expanding the adoption of the study-a-thon.

## WHAT IS A “STUDY-A-THON”?

**Figure 1.**
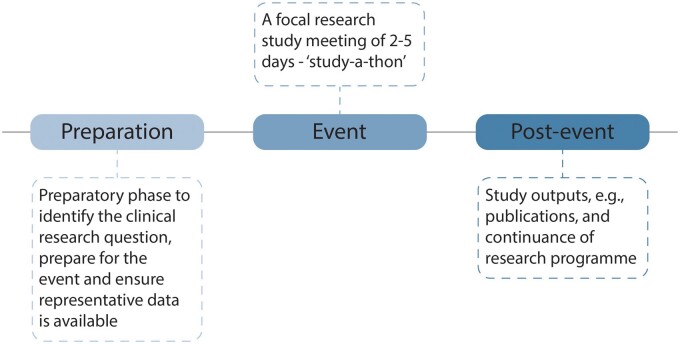
Outline of a new innovation, the study-a-thon.

Many researchers, especially those involved in working with data and application development or insights generation, are probably familiar with the concept of the “hackathon,” an organized event in which a large number of people meet to engage in collaborative computer programming. OHDSI study-a-thons can be seen to be different to hack-a-thons, with the former focusing on a clinical or research question, whereas the latter typically is aimed at specific technology challenges or topics. The research study-a-thon, as developed by OHDSI, incorporates a similar approach to a hackathon, bringing together a group of informaticians, data scientists, academic clinicians, epidemiologists, and patient representatives, to conduct observational studies on clinical issues of interest or priority over two-to-five intense days, depending on the scope and scale of the research, as outlined in Figure 1.

Critically, a study-a-thon is an opportunity to have focus for researchers with the required experience and skills, together in an organized research scrum with relevant data in one location, geographical or virtual, concentrating together on the identified research query.

To date a number of study-a-thons have been performed, within OHDSI, or in collaboration with the European Health Data and Evidence Network (EHDEN), an Innovative Medicines project that is building a federated network of data sources standardized to the OMOP CDM in Europe.^4^^,^^9^

Study-a-thons are characterized by their preparatory work including

- relevant colleagues with the most relevant experience and skills, as well as applicable knowledge, with
- Data Partners who are the custodian of OMOP CDM-mapped datasets,
- a research programme over 2–5 days with an expectation of being able to populate initial abstracts and/or draft manuscripts by the end event, and
- a continuance programme thereafter to ensure closure of the research and publication of generated evidence.

The OHDSI approach, and characterized also within a study-a-thon, is the concept of using dynamic versus static publication, with real-time outputs, for example, R Shiny applications. This supports dynamic publication but also reproducibility of the research to other datasets during or following the study-a-thon. Furthermore, all analytical codes and results are publicly available during and after the study-a-thon via GitHub or https://data.ohdsi.org.

## EXPERIENCE TO DATE WITH STUDY-A-THONS

The study-a-thon history has grown over recent years and increased in frequency, with a summary overview of an initial six as below (some exemplar study protocols are referenced):

1. 2017: An initial 3-day pilot, prototype live event at Columbia University, New York City, on comparative safety in rheumatoid arthritis.^10^
2. 2018: Between OHDSI and EHDEN, a 5-day live event held at the University of Oxford on estimation and prediction studies evaluating partial, unicompartmental versus total knee replacement, based on emulating the TOPKAT clinical study protocol.^11–13^
3. 2020: In collaboration between OHDSI and EHDEN, a 5-day live event held in Barcelona, Spain, with characterization, estimation, and prediction studies on the first-line therapy in newly diagnosed rheumatoid patients versus international clinical guidelines.^14^
4. 2020: a 4-day virtual event held online due to pandemic restrictions for characterization, estimation, and prediction on COVID-19 to assist with policy and clinical decision-making early in the first wave of the pandemic and then continue with ongoing characterization and estimation protocols.^7^^,^^15–17^
5. 2020: during the annual US-based OHDSI symposium a virtual, 2-day online event entitled PROTEUS on prediction external validation in cardiovascular care.^18^
6. 2021: a 5-day virtual event was facilitated by the IMI PIONEER project, in collaboration with IMI EHDEN, and OHDSI, to evaluate the real-world outcomes of the decision to initiate “watchful waiting” of prostate cancer progression when treatment is not indicated utilizing phenotyping, characterization, and prediction studies. This study-a-thon was also noteworthy for the level of patient participation to contextualize the research work from a patient experience perspective.^19^^,^^20^

The six study-a-thons have increased in size, the challenge of research conducted, and outputs and have provided considerable opportunity to refine the research process to conduct further research events. For the six study-a-thons, we have seen an escalation of activity, with an increasing number of publications, in particular related to the fourth, COVID-19 study-a-thon.

For the first study-a-thon, held in 2017, as a prototype, this was an experimental event to understand the feasibility of conducting rapid research utilizing the OMOP CDM and standardized analytical tools, incorporating approximately 40 participants and five OMOP CDM-mapped datasets. For the second study-a-thon, a resultant oral presentation at the EULAR conference in 2019 was accompanied by a publication in *Lancet Rheumatology*, in which the editorial comment stated, “Burn and colleagues are to be applauded for using innovative methods and adding to the evidence base, helping to better inform patients and surgeons in the shared decision making process.”^21^ For the third event, publications were submitted or close to following disruption by the pandemic with one article published and five abstracts presented in various conferences.^15^^,^^22–25^ Meanwhile the fourth study-a-thon has resulted in multiple publications on characterizing patients with COVID-19, the adverse event profile of hydroxychloroquine with or without azithromycin in a representative rheumatoid arthritis population, as well as public health prediction models. It was noteworthy that this COVID-19 study-a-thon included approximately 330 participants from 30 countries with 20 data sources, and the adverse event study contributed to the regulatory warnings of both the Food and Drugs Administration (FDA) and the European Medicines Agency (EMA).^15^ The fifth study-a-thon is completing outputs from the event held in late 2020, evaluating cardiovascular prediction modeling for short- and long-term risk, with a planned two publications. The sixth study-a-thon is working on outputs from its events held contemporary to this paper.

## CONSTRUCTING A STUDY-A-THON

**Figure 2.**
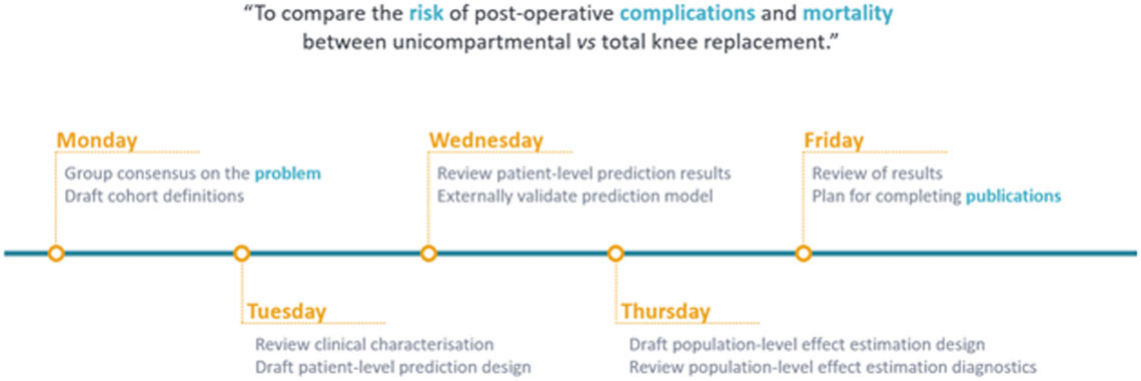
Outline of the second OHDSI study-a-thon and the typical content over 1 week. OHDSI: Observational Health Data Sciences and Informatics.

The study-a-thon programme typically includes activities for protocol development and cohort definition, followed by the implementation of characterization, population-level effect estimation, and patient-level prediction studies. Output milestones by study groups are held with feedback sessions at the start and end of each day, culminating in the formulation of abstract content at the least, or initiation of manuscripts by the final day. The outline of the second OHDSI study-a-thon in Figure 2 outlines the event-based schedule of the activities and dependencies over a week (5 days). For study-a-thons of shorter duration, activities may be truncated or performed prior to or on day one.

**Figure 3.**
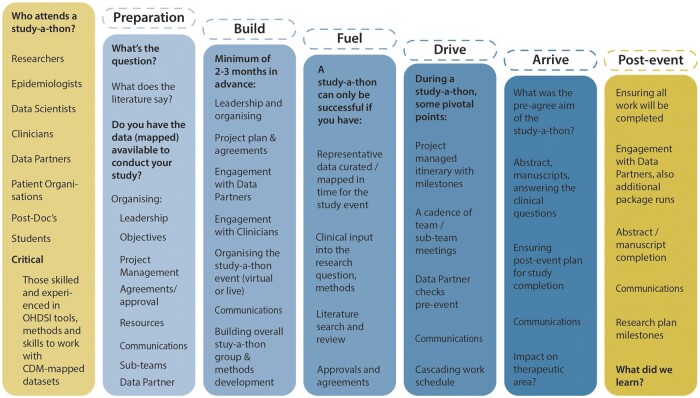
Outline flow of a study-a-thon.

Based on the experience of the six study-a-thons, a number of insights have been generated to optimally conduct future research events and to assist the wider research community with establishing their own study-a-thons, incorporating standardized data and standardized analytical tools, such as ATLAS.^4^ We will now outline the insights by relevant category, and as summarised in Figure 3:

- Leadership
  - It is important to have identified study-a-thon leaders, preferably also with project management support. This leadership team needs to guide all others in the study-a-thon, anticipating key steps in the workplan, coordinating, and managing the conducted research. This also needs to recognize that though the central focus of a study-a-thon is research, there are also education and community-building aspects to participating in one. The leaders need also to align participants’ skills, background, and preparation to activities in the study-a-thon workplan. In keeping the transparent and open nature of the study-a-thon, the leadership is integral to maintaining both the scientific and educational aspects at the core of this activity.
- Rationale
  - There needs to clear scientific rationale that contributes to the science and understanding of clinical issues, as well as responding to current evidence gaps. It needs to be transparent to all involved and with very clear outputs to communicate its findings. The decision to conduct a study-a-thon needs to be based on this being the best methodological approach to meeting the scientific aims and research need.
  - (A study-a-thon generally follows one of the three types of research questions, as further explained in their respective chapters in part IV of *The Book of OHDSI*^4^:
    - Characterization: characterizing populations through the use of descriptive statistics to generate hypotheses about the determinants of health and disease and to understand the clinical outcomes of specific groups in the population.
    - Population-level estimation: the estimation of average causal effects of exposures (eg, medical interventions such as drug exposures or procedures) on specific health outcomes of interest (eg, the safety or effectiveness of drugs or other treatments).
    - Patient-level prediction: the prediction of future health outcomes from existing patient-level data, to support clinical decision-making and risk evaluation and the validation of such prediction models.]
- Objectives
  - Need to be very clear in advance of the study-a-thon and provide the framework for all the points above, but also the ability to evaluate the success or key performance indicators (KPIs). KPIs ought to be closely related to the study protocol and proposed study outputs, and also any specific KPIs agreed by the study-a-thon team in advance for that specific event. Given the time, effort, and commitment required, a study-a-thon should aim at providing a significant contribution to scientific evidence and/or clinical practice. Though this is predominately scientific in nature, there will be other KPIs from relationship building to Data Partners, to socializing participants into, for example, the OHDSI framework. Realism needs to be at the heart of this in terms of what can be achieved, albeit with some stretch, but better in a study-a-thon to show results, than land too short.
- Preparation
  - The study-a-thon commences months prior in terms of ensuring everything from agreements to collaboration with Data Partners through to literature review. The success of the study-a-thon will be impacted by not being suitably prepared, and in essence, it further reinforces that the event is not actually the totality of 2–5 days, but the preparation and postphase too. The prior literature review informs the current knowledge and evidence gaps in the clinical domain of interest, contextualizing what has been performed and how helping with background for design and phenotyping decisions. Study leads can prepare the study-a-thon, initial outline, and project management planning and allowing for discussion on key elements requiring exploration and consideration in the proposed study.
- Team and sub-teams
  - To be most effective within a study-a-thon, consolidating the research workplan into smaller components that can be tackled by 3–10 participants can ensure also that tasks can be implemented, under the study-a-thon’s leadership. To achieve the study-a-thon’s goals, breaking down into subteams, covering literature review, clinical characterization, phenotyping (cohort definition), estimation and prediction studies (if applicable), study package development, and data sources support, works well. However, these topics are closely linked, and interdependent, so ample room should be provided to allow for communication between the groups during and after the study-a-thon. Also, the individual groups need equitable balance of knowledge and talent, so understanding participants’ skills, knowledge, and experience prior to the event (eg, via a survey) will help work out the team and subteam roles with the right mix (inclusive of the technical, methodological, and clinical skill mix). The size of the study-a-thon group historically depends on it being a physical or virtual event, with the former being between 40 and 50 people, and virtual being possibly three times this (though this increases the logistical, project management and leadership pressures).
- A suggested subteam organization
  - In a study-a-thon, four important groups or themes work in tandem to achieve the end result:
    - The literature review and clinical research group focuses on refining the proposed research question that is relevant, specific, and timely given the state of the art in medical research and the potential data contributors to the study-a-thon. The design needs to be well developed, if not finalized, before starting with, for example, phenotypes. Certain aspects such as events, period of study, and exclusion and inclusion criteria need to be well defined and written in the protocol before starting with building the cohorts.
    - The phenotype development group takes the research question (following earlier work incorporated into the protocol) and translates that into a number of specific cohort definitions, which can then be used to execute the study. This requires balancing the often elaborate wishes of the clinical research group in terms of characterizing populations and outcomes, with the actual data available in the participating databases.
    - The study execution group translates the research question into programming code (a “study package”), using the concept and cohort definitions developed by the phenotype development group. This also requires recruitment and coordination of all the databases participating in the study-a-thon. The study package is executed by participating databases, and the results are integrated and ideally published in a real-time in an R/Shiny app.
    - The management group focuses on facilitating the study-a-thon as a whole and the discussion on interpreting findings resulting from it (see Project Management below).
- Working with Data Partners
  - A study-a-thon will not be able to execute research without relevant, representative, and accessible data for the research question in mind. Approaching Data Partners, most likely with a pre-protocol synopsis for the study-a-thon, early is an imperative to ensure receptivity and engagement. The full protocol needs to be agreed with Data Partners and clinical and methods experts, predicated on the need by many for internal approvals and agreements. It is important to recognize specific data sources requiring prior approval for study conduct, and a study-a-thon is not a method to subvert this requirement. Meanwhile, assuming a prerequisite for a Data Partner’s dataset to be mapped to a CDM, allowing time to evaluate the mapping of the Data Partners’ dataset, running initial query scripts, and evaluating any technical issues prior to the study-a-thon would be invaluable in preparation. The real-time analysis of results by Data Partners on top of the CDM-mapped dataset considerably facilitates the ability to discuss the intended research focus individually and ensemble with the Data Partners. It also assists researchers with deeper insights into the data’s provenance, quality, and context, as well as emphasize the collaborative nature of the study-a-thon, its transparency, and common learning opportunity.
- Project management
  - Ensuring that some participants have the role of project management, for smooth running pre, peri and post, but also reinforcing KPIs and timeliness in terms of working to a prescribed agenda are critical—study-a-thons are of course a condensation of many months of normal timed activity in terms of days and it all needs to run like clockwork. Using the communications and collaboration platform is critical, such as MS Teams or others, which need to be set up and planned out in advance. Space to work, repositories, and communications in a virtual environment, even when a physical meeting, will enhance a study-a-thon. The well-worked out OHDSI framework for conducting studies already provides a rational basis for conducting a study-a-thon, inclusive of logical milestones that maintain forward progression, but being able to pivot and be flexible is important if problems, challenges, or unexpected results occur. This flexibility will be even more critical if working across multiple time zones, with focus on logistical details to ensure it all runs smoothly during the study-a-thon. Ensuring whether a physical and/or virtual event, breaks and off time need to be programmed in to ensure that despite the significant workload there are appropriate rest intervals.
- Longer-term goals
  - Assuming the process of a study-a-thon ends at the end of the physical/virtual meeting is likely naive. As well as preparatory work, postevent work needs to be not just about tying up loose ends, but also an ongoing scientific enquiry. If participants think that it is just the study-a-thon and also presume that it is about a paper(s) at the end, then contribution and participation will fall off markedly after the event, so knowing what the ideal critical mass of remaining participants to ensure success is, is important. There may also be a series of study-a-thons, like stepping stones or an agile process to consider over an extended time period.
- Socializing OHDSI
  - Study-a-thons can be a great opportunity to further socialize and expose the OHDSI framework within a condensed time and environment to participants, supporting further curiosity of learning more, as well as providing an opportunity for all to be impressed by what can be achieved (so long as realistic). Involving Data Partners, who know their own data best, and can be involved in running scripts and R Shiny applications, is important for the study-a-thon but also valuable in exposing them to the merits and utility of the CDM, as well as the standardized analytical pipeline. This is specific to OHDSI study-a-thons, but the principle of socialization of the research process can be applied more widely.
  - Of note is the increasing involvement of patients in study-a-thons, most significantly in the sixth study-a-thon, where patients were not just representatives, informing on their personal stories, but also being involved in the research process, priorities of focus, and helping guide researchers on relevance to them as, in this case, men living with prostate cancer or its aftermath.
- Education
  - Study-a-thons are excellent opportunities to educate through application, that is, active participation in conducting studies, whilst being a learning experience, in particular as results can be rapidly presented during one. Especially for the participants there needs to be time for formalizing insights, as well as a routine within the study-a-thon duration for bringing the team, and also subteams respectively together, ensuring alignment and everyone is aware of overall progress and any issues. Through subteams and individual time-conducting tasks and time, this further facilitates learning. This is a really condensed experience, inevitably requiring some prior knowledge to get the best out of it, but also meaning some aspects of the programme will be fast and challenging, reinforcing that need for a routine of the group coming together periodically. Noticeably, where there have been some introductory lectures by the study-a-thon leaders, this is very helpful, as well as familiarization with the topic area (clinicians important here to set the context) and the OHDSI framework via, for example, the EHDEN Academy (a free educational spin-out from the EHDEN project; https://academy.ehden.eu) and *The Book of OHDSI* (provided free online; https://ohdsi.github.io/TheBookOfOhdsi/),^4^ probably should be prerequisites too.
- Communications/dissemination
  - Effective communications are vital for internal management of the study-a-thon group, using platforms like MS Teams (others are available, eg, Zoom or Slack), pre, during, and post the event, but also externally communicating to key relevant stakeholders, and publication. Critically, the transparent and open science nature of a study-a-thon requires pertinent communications activity. Having ideas on target journals in advance will help too, as well as any editorial relationships within the group, and this will also inform the abstract(s) and/or manuscript(s) development with regards to format, etc., but also timelines and peer review thresholds. Communicating also needs to be seen with reference to expectation management, which means objectives communicated need to be met, if not for scientific rationale, but also for credibility.
- Finalizing
  - Does a study-a-thon really end? If it is seen as part of a research continuum, perhaps not, but even so a commitment to meet original goals and KPIs by the end of the event, but also a realistic timeframe following it to conclude publications, presentations, and communications, from weeks to months. Ensuring participants realize this does not necessarily end on a Friday afternoon is a key learning, so those invited need to understand they are committing to not a “5-day study-a-thon,” but a process still of perhaps many months.
  - A study-a-thon is a catalyst, focusing what would have been ordinarily a longer period into a shorter sprint, with both pre- and postevent. A unique feature of the study-a-thon is the concentration of multiple disciplines, working on a specific question, in parallel rather than sequentially. The commitment must be for the whole of this period in terms of a research study, incorporating a study-a-thon to meet the study aims in a reduced timeframe. This is a challenge for those returning from a study-a-thon to their daily workload and priorities, so a postperiod should also be project managed via the study-a-thon leaders with clear goals, milestones, and timelines to conclude the research and publication process. This has been a challenge in prior study-a-thons, but recent response to this, for instance in the PIONEER, sixth study-a-thon is providing dividends in post-follow-up activity and will be evaluated for future ones.
  - Being able to “return to the room,” or venue whether physical and/or virtual should be encouraged, taking participants back to the study-a-thon experience and reinforcing shared goals, as well as that sense of collaboration and enquiry. Utilizing online forums, such as MS Teams, can facilitate this at least virtually, and in using the original environment as the study-a-thon. This is especially important as there is always a risk that enthusiasm may wane following the study-a-thon, leaving a core of researchers to finalize any outputs, inclusive of publications.
  - Importantly, study-a-thons have led to further development and refinements within the OHDSI community more generally for tools and methods, inclusive of creation of focused working groups, such as for COVID-19.
- Resourcing
  - Do not underestimate costs, not just financial, but also for time, volunteering, logistics for a physical meeting (so accommodation, catering, travel), and ensuring all KPIs can be met pre, during, and post a study-a-thon. Most often, resources have come from within OHDSI, EHDEN Partners for the five study-a-thons in terms of “making it happen,” but alongside project management, resource planning will ensure smoother execution of the event at all stages, inclusive of outputs. Finally, a study-a-thon will be successful or not on the accessibility and availability of (CDM-mapped) data to ensure that the research output is both credible and representative of clinical reality, and the clinical domain and, for example, hospital and/or secondary care data availability will impact on the likelihood of success. The data need to “fuel” the study-a-thon “engine,” driven by the “engine” of the network of participants, their skills, knowledge, and experience, and “driven” to the destination of the event’s target research enquiry. Financial support for data partners, such as for data access fees, should also be incorporated as a contingency.

## DISCUSSION, FUTURE PERSPECTIVE, AND RECOMMENDATIONS

The study-a-thon as a research process, outlined in Table 1, has been shown to be an effective method and process for conducting rapid observational health research whilst maintaining quality of the science and its results, especially in response to the COVID-19 pandemic.^7^^,^^15^ The OHDSI community will be working to establish further study-a-thons as a unique approach, responding to public health and clinical priorities, and assisting the research community in conducting them.

**Table 1.** Summary outline of a 5-day study-a-thon activities by day

Pre	Day 1	Day 2	Day 3	Day 4	Day 5
Problem definition	Group introductions, subgroup iteration, and logistics reiteration	Review clinical characterizations	Review patient-level prediction results	Draft population-level effect estimation design	Review of results and agreement on continuing work requirements
Pre-protocol/synopsis definition	Problem definition (unless defined prior)	Draft patient-level prediction design	Externally validate prediction models	Review population-level effect estimation diagnostics	Post-study-a-thon planning and commitment
Literature review planning and initiation	Refining research question and protocol finalization	Iteration of phenotypes, cohort definitions, and study-a-thon “library”	Iteration of phenotypes, cohort definitions, and study-a-thon “library”	Abstract(s) development and initial manuscript outlining	Plan for completing publication(s)
Data Partner engagement and initial OMOP CDM mapping evaluation and/or ETL cycle for de novo mapping to CDM (with evaluation)	Draft cohort definitions and phenotype development (based on finalized research question)	Review by clinical experts and evaluation of results (vs clinical expectations)	Review by clinical experts and evaluation of results (vs clinical expectations)	Review by clinical experts and evaluation of results (vs clinical expectations)	
Logistics planning for in-person, virtual, or hybrid study-a-thon	Literature review completion/ongoing support for evidence (protocol support)	Study package run by Data Partners with ongoing refinement/issues and error support	Study package run by Data Partners with ongoing refinement/issues and error support	Study package run by Data Partners with ongoing refinement/issues and error support	
Licensing, agreements, contracts, and approvals (oversight of Project Management)	Study package encoding (based on concept and cohort definitions)	R Shiny Application for results sharing and QC	R Shiny Application for results sharing and QC	R Shiny Application for results sharing and QC	
Readiness agreement for study-a-thon commencement (in good time)	Leadership and Project Management check in with study-a-thon group/communication summary	Leadership and Project Management check in with study-a-thon group/communication summary	Leadership and Project Management check in with study-a-thon group/communication summary	Leadership and Project Management check in with study-a-thon group/communication summary	

CDM: common data model; ETL: Extract, Transform, Load; OMOP, Observational Medical Outcomes Partnership; QC: Quality Check.

Other initiatives have also utilized approaches to conducting more rapid research processes, such as hack-a-thons or crowdsourcing, for instance, the AllBio project in bioinformatics, or the Dialogue on Reverse Engineering Assessment and Methods (DREAM) Challenges open science, collaborative challenge framework. For the AllBio, “Ten Rules” has some similarity with the OHDSI study-a-thon experience described here, and both provide a framework from “bio-hackathons” to observational research study-a-thons. The DREAM Challenges approach is more focused on a crowdsourcing strategy, driven by a global call, but local teams developing solutions, a very different proposition and process to OHDSI study-a-thons or AllBio hackathons. Inherent in the DREAMS Challenges is a legitimate competition driver,

For the demands in health care, and for the increasing expectations for answers in the short term that can inform and impact on clinical care and patient outcomes, innovation in how we conduct research utilizing RWD is critically needed. Being able to incorporate all key actors, as well as harmonized data from an international collaboration, whilst utilizing a logical approach with standardized analytical tools, and educating whilst researching, all in a specific timeframe has demonstrated clear benefits.

Further study-a-thons are planned, and the programme will be expanded as the OHDSI and EHDEN networks similarly grow to support observational health research in the 21st century.

## FUNDING

Research conducted and discussed was in part funded via The European Health Data & Evidence Network (EHDEN), which has received funding from the Innovative Medicines Initiative 2 Joint Undertaking (JU) under grant agreement No 806968. The JU receives support from the European Union’s Horizon 2020 research and innovation programme and EFPIA. This funding mechanism applies to the authors PRR, KvB and DP-A.

## AUTHOR CONTRIBUTIONS

NH was a lead author and drafted the initial outline, and PRR, KvB, TD-S, CS, DV, DP-A, and PR all contributed equally on editing and review of the drafts, and subsequent revisions, as well as final approval for submission, all as per ICMJE guidelines.

## CONFLICT OF INTEREST STATEMENT

NH and PR are employees and stockholders of Janssen and Pharmaceutical Companies of Johnson & Johnson. PRR works for a research institute who receives/received unconditional research grants from Yamanouchi, Pfizer-Boehringer Ingelheim, GSK, Amgen, UCB, Novartis, Astra-Zeneca, Chiesi, and Janssen Research and Development, none of which relate to the content of this work. KvB and TD-S report no conflict of interest. CS receives fees for project management from Bayer AG. DV is an employee of Bayer Pharmaceuticals. DP-A leads a research group, which has received grant support from Amgen, Chiesi-Taylor, Novartis, and UCB Biopharma. His department has received advisory or consultancy fees from Amgen, Astellas, Astra-Zeneca, Johnson and Johnson, and UCB Biopharma and fees for speaker services from Amgen and UCB Biopharma, none of which relate to the content of this work.

## Data Availability

There are no new data associated with this article (No new data were generated or analysed in support of this research).
